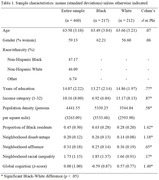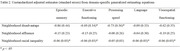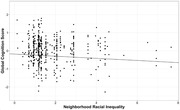# Neighborhood racial income inequality and ADRD risk

**DOI:** 10.1002/alz.090988

**Published:** 2025-01-09

**Authors:** Laura B. Zahodne, Ketlyne Sol, Kiana A. Scambray, Ji Hyun Lee, Jordan D Palms, Emily P. Morris, Lauren Taylor, Vivian Ku, Mary Lesniak, Robert Melendez, Michael R Elliott, Philippa Clarke

**Affiliations:** ^1^ University of Michigan, Ann Arbor, MI USA; ^2^ Montana State University, Bozeman, MT USA

## Abstract

**Background:**

Neighborhood conditions and their racial patterning represent under‐studied factors that could contribute to racial disparities in dementia risk. Neighborhood socioeconomic status (SES) has been linked to dementia, but the racial distribution of SES within a neighborhood may also matter for dementia risk.

**Method:**

Individual‐level data from 460 (47% Black, 46% White, 7% other) older adults from the Michigan Cognitive Aging Project (Table 1) were linked to census tract‐level data from the National Neighborhood Data Archive. Neighborhood SES was operationalized as composite measures of neighborhood disadvantage and neighborhood affluence. Neighborhood racial income inequality was operationalized as the ratio of median incomes for White versus Black residents. Generalized estimating equations accounting for geographic clustering examined associations between neighborhood factors and performance on a comprehensive neuropsychological battery, adjusting for individual‐level (age, gender, race/ethnicity, education, income) and neighborhood‐level (population density, proportion of Black residents) covariates.

**Result:**

Neighborhood racial income inequality was uniquely associated with worse cognitive health across multiple domains (Table 2, Figure 1), and these associations did not differ by participant racial identity. Neighborhood disadvantage was only associated with worse cognitive health among Black participants (*p*’s < .007).

**Conclusion:**

Both overall neighborhood SES and racial inequities in the distribution of economic resources within neighborhoods are associated with dementia risk independent of individual‐level SES and other characteristics of the neighborhood. Findings highlight potential negative cognitive impacts of structural racism on a community that may be universal. Racial differences in both the level of neighborhood disadvantage and the strength of its associations with cognitive health (i.e., differential impact) likely contribute to racial inequalities in dementia. Future work should characterize mechanisms underlying these effects, as well as develop and test policies and interventions to improve neighborhood conditions and dismantle structural racism in order to promote health equity.